# Complex inner and outer setting interactions determine feasibility and readiness of developing primary care registries in small island developing states: sequential mixed methods study

**DOI:** 10.3389/frhs.2025.1593902

**Published:** 2025-09-30

**Authors:** Natasha P. Sobers, Joeleita Agard, Jacqueline Campbell, Kia Lewis, S. M. Jeyaseelan

**Affiliations:** ^1^ George Alleyne Chronic Disease Research Centre, Caribbean Institute for Health Research, University of the West Indies, Bridgetown, Barbados; ^2^ Glasgow-Caribbean Centre for Development Research, The University of the West Indies, Bridgetown, Barbados

**Keywords:** registry development, pre-implementation phase, feasibility assessment, noncommunicable diseases, small island developing states

## Abstract

**Introduction:**

We assessed feasibility and readiness for registry development and sought to understand the factors likely to affect the implementation of a registry into health systems in small island developing states (SIDS).

**Methods:**

We conducted online quantitative surveys and focus groups among health managers in four SIDS. Both survey and focus group guides were developed primarily based on four domains (inner setting, outer setting, individual characteristics and implementation process) of the Consolidated Framework for Implementation Research (CFIR). Focus groups within each of four territories were recorded, transcribed verbatim and analyzed using thematic content analysis using a deductively derived coding framework. We synthesized our findings using a systems archetype informed by the common themes emerging from the dual methods.

**Findings:**

From the 37 respondents of the online survey, 40% and 16% identified diabetes and hypertension as the highest priority NCDs for registry development. Respondents were more concerned about monitoring and improving care for patients (65%) than about determining disease burden (16%) or outcomes monitoring (8%). Strong mission alignment, external pressure and support and emerging data protection laws were identified as potential facilitators. Participants reported lack of human resource capacity and funding to support NCD registry and poor information systems infrastructure. The emerging systems archetype indicated that lack of investment in human and systems infrastructure were significant threats to registry success.

**Discussion:**

Despite external interest in registry development, infrastructural and human resource capacity barriers are likely contribute to a sub-optimal implementation in SIDS. We recommend greater collaboration between countries and enhanced regional support to overcome the challenges.

## Introduction

Small island developing states (SIDS) account for a disproportionately high burden of non-communicable diseases (NCDs) (1). Recognizing this, governments in SIDS have made several political commitments to enact policies and programmes and restructure healthcare systems to facilitate the cost-effective prevention and management of NCDs (2).

Monitoring progress towards the fulfilment of these policies, requires routine, systematically collected data. One of the challenges for SIDS has been in the collation and use of local data to inform policy and decision-making (3). A World Health Organization (WHO) brief describes health information systems in SIDS as “weak, underdeveloped and fragmented” (4).

Patient registries provide a solution to this issue, as they systematically capture, collate and disseminate data that can be used to understand burden of disease, real-world treatment and management practices (5). They form a key component of the quality improvement cycle in the healthcare system, informing the planning and actions of health system stakeholders (6, 7). Despite their usefulness in enhancing care, primary care registries are uncommon in the Caribbean and other SIDS.

For successful registry planning, careful consideration must be given to various factors including the available resources, registry purpose and scope (5). Several generic and disease specific publications described the planning and implementation of a registry (8–10), and others have assessed factors influencing successful implementation (11–13).However, none of these used an implementation science framework to examine the pre-implementation/planning phase of registry development and none occurred in SIDS.

Further review of the literature revealed that studies often described linear connections between the registry factors, acknowledging but not elucidating complexity. A systematic review of implementation science frameworks and systems science methods reported that few studies explored potential synergies in both approaches and called for more work to consider how they may be aligned (14), since it is unclear on how best to combine these methods. We theorize that mapping the interconnectedness of the systems surrounding registry development may better inform sustainable implementation.

Specifically, the pre-implementation phase of registry development includes assessments of feasibility and readiness (15). In the study, feasibility refers to the presence of critical factors required for successful registry implementation and readiness refers to the factors which are in place or about to be so in the setting the registry is to be implemented.

We aim to explore the feasibility and readiness of implementing primary care patient registries in select SIDS using the Consolidated Framework for Implementation Research (CFIR) and systems mapping. Through this, we also intend to understand the interaction of the factors associated with feasibility and readiness from the perspective of government and facility level stakeholders. A sequential mixed methods design was chosen to conduct a quantitative feasibility assessment to guide the selection of the SIDS for the readiness assessment, which includes both qualitative and quantitative methods. This approach aids in understanding the nuances in the facilitators and barriers to registry implementation in varying contexts.

## Methods

### Study design and setting

The United Kingdom Overseas Territories (UKOTs) are a group made up of fourteen small island developing states which have constitutional links with the United Kingdom. UKOTs are self-governing with healthcare systems being managed by local governments with technical and financial support from the United Kingdom (UK). Their population of citizens range from under 5,000–64,000 but there is significant variation in numbers of ex-patriates and seasonal residents. Invitations to participate in this study were sent to all UKOTs with a permanent population.

### Participant selection

This study received ethical approval from the University of the West Indies/Ministry of Health and Wellness ethical review board (Ref: CREC-CH.0017403/2023) as well as approval from the responsible authorities in each participating territory. After permissions were obtained, a link to the feasibility survey was sent to the territory focal point for circulation to the relevant staff. Relevant staff were identified by the territory team and could either be involved with management of NCDs (e.g., at a facility level), and/or infrastructure (e.g., electronic health records, information technology support).

Participants were selected based on their expertise and roles in their relevant healthcare systems. While the identification of the participants and key informants was conducted by the ministries, it was the researchers who approached the participants to explain the study, provide information and request consent (to reduce the risk of coercion). Participants were asked to review study information and complete consent forms for both survey completion and participation in focus groups. To preserve anonymity, consent was indicated by ticking a series of boxes in response to questions. Participants were also offered the option of taking part in the assessment to inform the registry development but not have their results included in the research write-up. All participants in the focus groups consented to being part of the research project. Individuals who met any of the following criteria were invited to take part: (1) Healthcare professionals (Clinical experts) involved in population treatment and management of NCDs, (2) Policy makers involved in decision making around registries, (3) Public Health professionals and epidemiologists and (4) Information Technology specialists responsible for database management and electronic health records.

### Data collection tools

#### Quantitative data collection—online survey

Quantitative data was collected using an online form using REDCap electronic data capture tools (16, 17) hosted by CaribData for The University of the West Indies (UWI), a project funded by the Inter-American Development Bank (IDB). The survey link was sent to Non-Communicable Disease (NCD) and surveillance stakeholders identified using the participant selection criteria above. The participants from the qualitative focus groups were chosen from the same participant selection pool as the online survey.

We developed the framework for the registry feasibility and readiness assessments by reviewing and identifying relevant constructs of the Consolidated Framework for Implementation Research (CFIR) 1.0 (18) (Supplementary Table S1). We operationalized select domains of the framework in the following manner:

**Intervention characteristics—**The core characteristics of the planned disease registry, without being adapted to a specific context
**Characteristics of Individuals—**The interplay between individuals and the potential effects on implementation of their teams, units and networks
**Inner Setting**—Ministry of health/health authorities, primary care clinics, Hospitals (secondary and tertiary care facilities), private care
**Outer Setting**—All agencies external to the Ministry of Health, healthcare facilities, staff and managers

The purpose of the feasibility assessment, broadly distributed online survey, was to assess whether factors critical for successful registry implementation were in place. Factors were initially derived based on definitions within the CFIR framework (Supplementary Table S2). Following this, additional domains were added from the Physician Orders for Life-Sustaining Treatment (POLST) registry readiness assessment (19) and the Agency for Healthcare Research and Quality (AHRQ) user's guide for registries for evaluating patient outcomes (5) (Supplementary Table S2). Additional constructs were included based on our experience of registry implementation and health data collection in SIDS and the findings of Lazem et al. (20). The online survey also assessed elements of readiness. A focus group was then conducted which further explored elements of feasibility and readiness initially answered in the survey.

The link to the feasibility assessment quantitative survey was distributed via email to staff who met the inclusion criteria. The data from the online surveys was collected and stored in REDCap which enables data to be collected in a HIPAA and GDPR compliant environment. We developed feasibility and readiness scores based on the responses given. Supplementary Table S3 describe how these scores were developed.

#### Qualitative data collection—focus group

Having reviewed responses from the online survey, we developed semi-structured interview guides for the focus groups to be held with individual countries to better understand the responses given in the quantitative survey. Semi-structured questions focused on further assessing feasibility and readiness were used to guide the focus groups which were conducted virtually via Zoom and were attended by a group of potential registry implementers/stakeholders The participants within each country were generally known to each other since they all work within the government ministry or facility which will be responsible for implementation and have worked with each other in the past on similar projects. The participants from each territory (focus group) included an individual from each of the groups identified in participant selection. The group included Chief Health Planners, policy makers, Nursing Supervisors, Epidemiologists and Managers of Information Technology/Health Information Systems. There was a tendency for physicians within the groups to speak first and more often than other participants. NS and SJ facilitated the focus groups and were generally unknown to participants before the project. Both NS and SJ consistently asked for feedback from participants who appeared to participate less in the questions, particularly when questions were specific to their area (e.g., Case of electronic medical records). NS and SJ are female public health researchers who work primarily in academia, but both with extensive experience working with fragmented data systems which often exist in developing countries. NS is from a small island developing state in the Caribbean while SJ is a British national, traits that may have made interviewees predisposed to working with one or the other”.

We initially sought to hold six focus groups with six to ten persons each but two of the territories were unable to obtain ethical approval and convene a meeting with stakeholders before the funder-imposed deadline for the project report. Our purposive sampling and focus group data collection was driven by the need to understand each health system enough to decide whether a chronic disease registry development was feasible and not by data saturation. However, in alignment with the work of Hennink et al, we believe four focus groups of five to eight persons each was enough to allow us to reach data saturation (21).

These sessions were audio-recorded and transcribed verbatim and used to enhance understanding of the responses provided in the online survey and only participants and researchers were present. Groups varied in size from five to eight. Each group was interviewed once, each session lasted an average of 59 min; notes were taken during the interview by the project team (JA).

#### Confidentiality

No personal data were collected as part of the quantitative or qualitative data collection processes. Participants within each focus groups were work colleagues thus voice recognition was likely. In consenting, confidentiality by researchers was guaranteed while participants were asked to maintain confidentiality of each other's responses. Given the information being shared was not sensitive and related generally to work matters and discussion between colleagues was thought to be likely to enhance information obtained, focus groups were considered appropriate. We have chosen not to report the responses of the focus groups as a group without identifying individuals to preserve confidentiality. In small populations, providing the roles would risk identification of the individual in reporting. Additionally, to prevent identification of participants, responses collected through the online survey were anonymous. Only the territory of the participant was collected as this is relevant to the assessment of feasibility and readiness. The audio files and transcriptions from the focus groups were encrypted (using AES-256 encryption standard) and stored on a password protected device. Only authorized members of the research team have access to audio recordings.

#### Analysis- quantitative and qualitative

Descriptive statistics (proportions, ranges and medians) were used to summarise territory readiness and feasibility. Four researchers (NS, JA, SJ, JC) discussed the preliminary findings of the quantitative survey and used this to guide the questions asked in the focus groups. Transcripts from the focus groups were coded using induction and analysed using a framework analysis. NS and JA developed the coding frame (Supplementary Table S4) using CFIR theory-based framework and induction. JA, NS and KL coded the transcripts, using NVivo version 12. JA created the matrix in Microsoft Excel and shared with the research team for verification and discussion of emergent themes. The co-authors met weekly to clarify the coherence and relevance of emergent themes across the transcripts. Transcripts were not shared with focus group participants for verification. In developing the conceptual model emerging from the themes, the research team used the systems archetypes developed by Kim et al. (22) to create a causal loop diagram using both the quantitative and qualitative findings. JA, KL and NS are trained in qualitative research at post-graduate level and NS has over five years' experience with studies and publications in mixed methods research. SJ and JC are quantitative researchers with strong positivist approaches.

## Results

### Intervention (registry) characteristics

Four countries completed both the quantitative and qualitative assessments. Focus groups consisted of eight, seven, five and eight persons from four territories. From the 37 respondents of the online survey, most respondents identified diabetes (40%) and hypertension (16%) as the highest priority NCDs for registry development (Table 1). Respondents were more concerned about monitoring and improving care for patients (65%) than about determining disease burden (16%) or outcomes monitoring (8%). None of the participants within countries were unanimous with respect to the priority disease and in only one country did respondents all agree that monitoring and improving care was their primary purpose. During the focus groups when stakeholders were engaged together, the participants in three territories reached general consensus on the disease of interest while in one case there was still disagreement on whether cancer or hypertension were the more important given the country profile.

**Table 1:** Survey responses by territory and selected domain of the Consolidated Framework for Implementation Research.

Questions within CFIR Domains	C1 (*n* = 8)			C2 (*n* = 8)			C3 (*n* = 10)			C4 (*n* = 10)		
Domain: intervention characteristics												
Primary Disease (Top 3)	Hypertension (4)	Diabetes (2)	CVD (2)	Cancer (4)	Diabetes (3)	Hypertension (1)	Diabetes (3)	Cancer (3)	Hypertension (1)	Diabetes (7)	Obesity (2)	Chronic Kidney Disease (1)
Purpose	Monitoring and Improving Care (8)			Monitoring & improving care (4)	Disease burden monitoring (2)	Outcomes monitoring (2)	Monitoring & improving care (4)	Disease burden monitoring (3)	Indicator tracking (2)	Monitoring & improving care (8)	Disease burden monitoring (1)	Outcomes monitoring (1)
Utility of a registry for improving healthcare	Range 85–100	Median 93.5		Range 70–100	Median 93		Range 75–100	Median 94		Range 79–100	Median 91	
Confidence in successful registry implementation	Range 50–100	Median 89		Range 34–97	Median 80.5		Range 50–90	Median 75		Range 10–100	Median 60	
Domain: individual characteristics												
Access to epidemiologist	Yes = 6	Unsure = 2		Yes = 7	No = 1		Yes = 10			Yes = 2	No = 5	Unsure = 3
Location of epidemiologist	Internal = 6			Internal = 7			Internal = 7	External = 3		External = 2		
Has a lead been identified	Yes = 3	No = 2	Unsure = 3	Yes = 3	No = 3	Unsure = 2	No = 1	Unsure = 9		Yes = 2	No = 2	Unsure = 6
Domain: inner setting												
IT management	In-house = 3	Externally = 2	Unsure = 3	In-house = 6	External = 1	Unsure = 1	In-house = 2	Externally = 3	Unsure = 5	In-house = 6	Externally = 3	Unsure = 1
Internet reliability	Very reliable = 3	Reliable = 3	Not reliable = 2	Very reliable = 3	Reliable = 3	Not reliable = 2	Very reliable = 2	Reliable = 4	Not reliable = 3	Reliable = 6	Not reliable = 4	
Internet connection speed	Very fast = 4	Fast = 2	Average = 2	Very fast = 3	Fast = 1	Average = 4	Very fast = 1	Fast = 4	Average = 4	Average = 7	Poor = 3	
EHR/EMR system	Yes = 8			Yes = 8			Yes = 6	No = 3		Yes = 9		
Which levels of health care have EHR/EMR?	Primary = 7	Secondary = 7	Tertiary = 2	Primary = 7	Secondary = 7	Tertiary = 3	Primary = 3	Secondary = 4	Unsure = 2	Primary = 8	Secondary = 6	Tertiary = 2
Do they all use the same system?	Yes = 7			Yes = 2	No = 4		Yes = 3			Yes = 2	No = 4	
Data extraction from EHR/EMR?	Yes = 6	Unsure = 2		Yes = 7	No = 1		Yes = 4	Unsure = 2		Yes = 5	No = 2	Unsure = 2
EHR searchable by diagnosis	Yes = 3	Unsure = 4		Yes = 6	Unsure = 2		Yes = 1	Unsure = 5		Yes = 1	No = 5	Unsure = 3
Registry form incorporated into EHR	Yes = 4	Unsure = 4		Yes = 4	Unsure = 3		Yes = 1	Unsure = 5		Yes = 2	Unsure = 7	
EHR linked to prescription data	Yes = 5	Unsure = 1		Yes = 8			Yes = 2	Unsure = 4		Yes = 9		
Physical space	Yes = 6	No = 1	Unsure = 1	Yes = 6	Unsure = 2		Yes = 5	Unsure = 5		Yes = 4	No = 3	Unsure = 3
Time	Yes = 4	Unsure = 4		Yes = 7	Unsure = 1		Yes = 6	Unsure = 4		Yes = 2	No = 2	Unsure = 6
Financial resources	Yes = 1	No = 2	Unsure = 5	Yes = 4	No = 2	Unsure = 2	Yes = 4	Unsure = 6		Yes = 2	No = 2	Unsure = 6
Domain: outer setting												
Any hard-to-reach populations	Yes = 3	No = 3	Unsure = 2	Yes = 6	No = 1	Unsure = 1	Yes = 3	No = 1	Unsure = 6	No = 7	Unsure = 2	
Proportion of hard to reach *	Range NA	Median 32		Range 12–83	Median 30		Range 11–26	Median 25		No response		
Who are hard to reach	Those seeking care overseas			Latinx	From private clinics or seeking care overseas	Undocumented patients	Those who do not access public health care services	Persons who attend private doctor or seek medical assistance overseas		No response		
Policies preventing EHR data sharing	Yes = 2			Yes = 2	No = 6		Yes = 1	Unsure = 6		Yes = 5	No = 3	Unsure = 2
Proportion of PPHC	Range 15–50	Median 27.5		Range 50- 80	Median 63		Range 0–50	Median 20		None		
Private hospitals (PH)	Yes = 4	No = 4		Yes = 7	No = 1		No = 10			No = 10		
Ease of data collection from PH	Range 2–4	Median 4		Range 1–5	Median 3		Not applicable			Not applicable		
Private pharmacies (PP)	Yes = 8			Yes = 8			Yes = 10			No = 10		
Ease of data collection from PP	Range 3–4	Median 3		Range 2–5	Median 3		Range 1–5	Median 3		Not applicable		
Private laboratories (PL)	Yes = 8			Yes = 8			Yes = 10			No = 10		
Ease of data collection from PL	Range 2–4	Median 3.5		Range 1–5	Median 3		Range 1–4	Median 3		Not applicable		
Need for external technical support	Yes = 8			Yes = 6	No = 1	Unsure = 1	Yes = 10			Yes = 9	Unsure = 1	
Importance of technical support	Range 5–10	Median 9		Range 1–9	Median 7		Range 5–10	Median 8		Range 6–10	Median 8	

### Characteristics of individuals: roles in and readiness for the registry

#### Project lead

The quantitative assessment indicated that while some reported that a lead had been identified, others were unaware or unsure of this. During the focus groups, all territories identified a potential lead but most of the leadership discussion centred around the political support which varied by country. In three of the territories political support was reported to be strong.

“I mean, we've been clamouring for that at the highest political level …. I believe, as a highest level of desire to have our data information collected”. C1

However, in at least one territory political support for registry development was reported as weak as it was thought that there was a lack of understanding of its importance.

#### Insufficient human resources

The survey showed that all territories had internal access to an epidemiologist- a person who should have the basic skills of registry science to support implementation. Group discussions focused on the staff needed to support the registry. Human resources were described as insufficient to meet the perceived increasing demands of a registry. Staff were described as already feeling overwhelmed by increasing daily tasks and lack of analytical capacity to integrate the electronic health records into monitoring care. Therefore, introducing a registry and producing data to inform policy may lead to frustration.

“Well, the workload for some persons it's tight, so that could be an issue ………… I'm also concerned that the staff may feel a tad bit overwhelmed because, even though we are in two years with this system, we are still struggling” C1

“For me, one of the biggest barriers (is), analytical capacity. Yeah, maybe I'm the only one in in, in the organization doing that. So that's one of the big barriers”, C3

#### Need for training

Most respondents (90%) identified the need for external technical support for the development of the registry. This was strongly confirmed in the qualitative data where all groups interviewed indicated the need for significant training in all the areas highlighted by interviewers. Data entry and coding were highlighted as priority areas for training in several territories, as this was thought to significantly affect data quality and its usefulness.

“One other thing. In terms of the coding, and so on. I think that there needs to be some more training, widespread training for persons who are doing coding”.- C2

“And you know they say garbage in garbage out and so sometimes the information that you're getting out of the data you're getting, it's not very usable. So it's important that persons who are doing the coding and we're ensuring that the information is properly inputted that those persons are trained properly”.- C2

In one of the territories the staff turnover was particularly high, due to having contracted workers from other countries temporarily fill roles. The importance of ensuring that someone based locally could be trained and then train others was emphasised.

“It would be helpful to have more than just John1 and Jane1 again, because we're contracted staff. So I just want to have somebody locally that could do some of this work as well”.- C4

### Inner setting

#### Culture: shared values related registry importance

In almost all the territories the establishment of a registry was reported to have good support among the workforce as persons understood the importance of collecting data to inform healthcare decisions.

“I would just want to say that there is some strength in the fact that our people want answers, and having a registry will give us answers. It will give us data … I think that is also a driving force and a strength that will enable persons to want to be a part of it and to cooperate with the process”.- C2

Across all territories they expressed optimism and support for registry development while recognizing the need to optimize their health information system to improve management of NCDs*.*

“We all see the need for it, I think, like on board, in trying to get the registry up on going, because we see where we flawed in the sense of not having a handle on Primary care as it relates to NCDs”. C1

#### Software/electronic health records (EHRs)

Both quantitative and qualitative data indicated that electronic health records are used to varying degrees in all territories but usually in parallel with paper-based records and implemented through vertical programmes. EHRs were generally searchable by diagnosis, linked to prescription data with the capability to incorporate registry forms. Some territories also noted good IT support with sufficient security to protect data.

“The IT team we have a quite strong system that I mean they're constantly evolving and ensuring that we're up to date with the latest levels that we're supposed to be at. And in terms of security. It's quite secure as well. That's that. That is an important component to any registry that you have, that security and the safety of the data that you're collecting” -C2

Even countries with electronic health information systems reported that the development of a new registry is likely to be a burden on existing human resources, so they recognise the need for the development of a team to advance these initiatives.

“I think the biggest issue we have is the fact that we can't autoextract …. it's going to end up on somebody's lap you know, every month to look at the diabetes information and if we expand this out to other non-communicable diseases and then it's going to be we'll end up having to have a team”. C4

#### Available hardware infrastructure

In most cases, healthcare workers had access to laptops or desktop computers, while the use of tablets and cell phones were more limited. In one country, it was noted that the epidemiology unit was ill-equipped with hardware necessary to manage a registry.

“Right now, the epidemiological unit is bare bones. I don't have a computer I like, I don't have the necessary equipment that I would need to manage the registries.- C2”

In another territory, it was noted that internet capabilities were limited, leading to concerns about the feasibility of an online registry system.

“So let's just say at present, our Internet capabilities aren't great”- C4

#### Variable financing support for registries in the inner setting

Only 30% of respondents indicated there was sufficient financial resources for the registry. Most participants were unsure of the availability of this resource. This was clarified in the focus groups. One territory reported that financial resources had been allocated for registry development, signalling political will.

“There definitely has been interest articulated over the years. That interest has I think, at times almost translated into resources and money being assigned ……. Some money has been assigned in the past, so I mean, I would think that I don't think there is an issue with political will to support”. -C4

In contrast, this was not the case in the territory in which there were concerns that adequate finances would not be allocated towards an NCD registry.

“People actually wanting to invest their time and money into this, yeah. I think that's the biggest barrier we have yeah … you can work around everything else but the fact that not everybody has the good intention to make this work. I think a lot of people see this as a problem and spending money and they might block it out”.- C3

### Outer setting

#### Influence of outer setting on inner setting priorities

**Figure 1:**
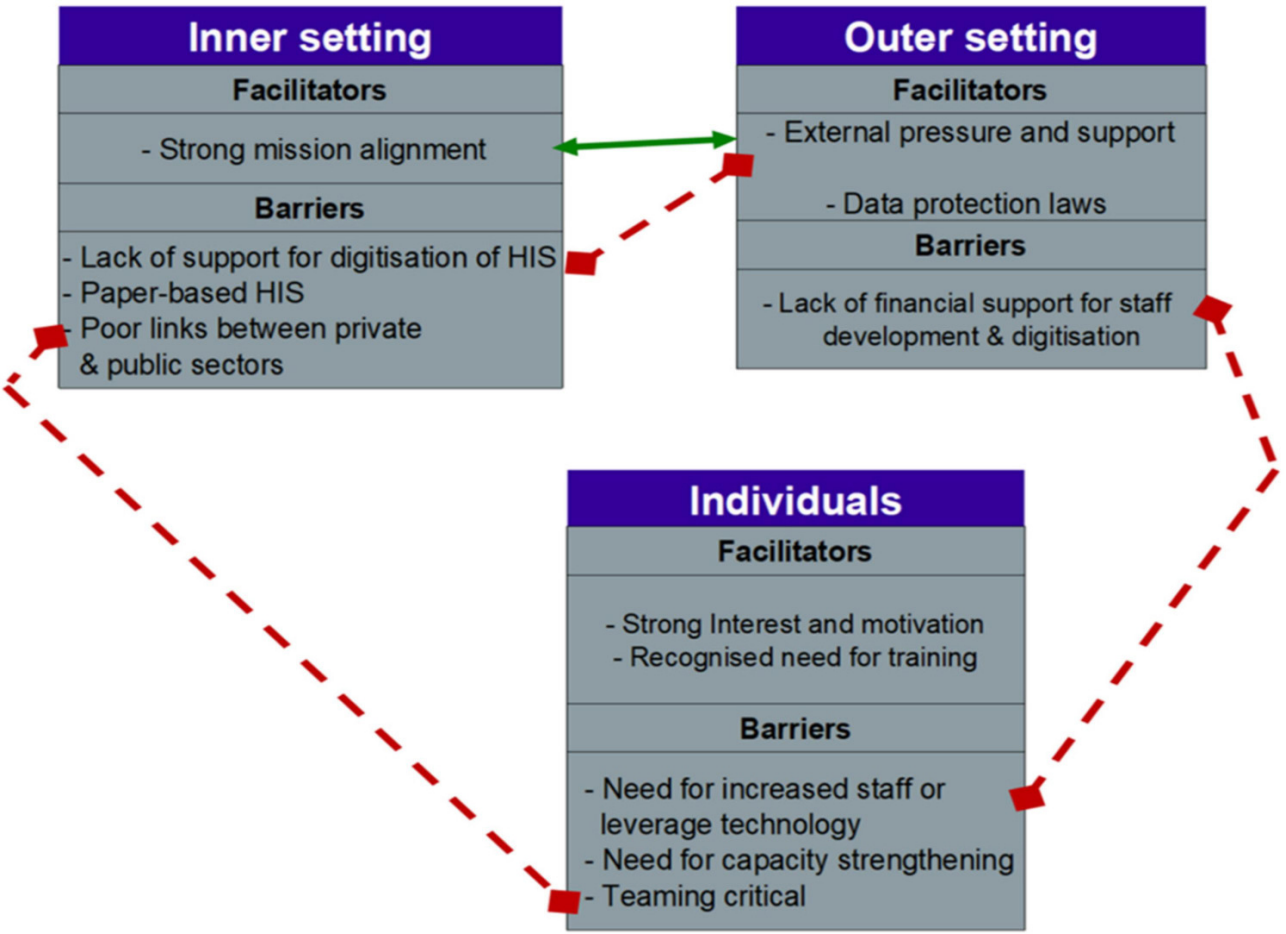
Facilitators and barriers interact in the inner and outer settings.

The political directorate within territories have been impressing on ministries the importance of developing registries to provide evidenced-based data to guide policy decision-making (Figure 1). Three of four countries reported “high levels of support” from government level while two out of four indicated that there were also high levels of support within the ministry and at the facility level.

“So I think it's(registries) been presented at [highest government decision making body]. And it's agreed that it's a way forward for the island”. C4

“…we've been clamouring for that at the highest political level they want it, and they'll be happy to know how and if and how it can be done”. C1

#### Support of outer setting for inner setting initiatives

In addition to support from the UK Health Services Authority (UKHSA), regional organizations like the Pan American Health Organization (PAHO) and the Caribbean Public Health Agency (CARPHA) have worked collaboratively with territories in the development of country level strategic priority and action items for the health sector that are in alignment with global and regional priorities. These priorities include reorienting health systems towards provision of high-quality data to aid in decision-making and the outcome documents provide some pressure to inner and outer setting actors to implement agreed upon initiatives.

#### Communications challenges between inner and outer settings

Challenges with communication between private and public sector were also acknowledged. It was thought that new communication systems would need to be put in place to properly facilitate this if private sector health facilities were included in the registry.

“Well, one of the challenges I would say that communication would probably be one of the biggest challenges. We would have to establish communication channels both up and down and across…how we would share information up the sectors, how we would share information between public and private, ……… and of course agreeing on the tools that would be used”- C2

#### Outer setting legislation influencing inner setting

With regard to the policy environment in most territories, legislation governing data sharing, privacy and security was not fully enacted.

“There's a whole suite of legislation that went through the process. I think it may have been passed, but not enacted as yet so presently at this stage as I said, no data sharing agreements between entities no enacted or active data protection and security legislation”.- C2

There was also no legislation mandating reporting for NCDs. Some participants thought this was a threat to data collection as the creation and enforcement of legislation mandating reporting was seen as critical to this process. Legislation was suggested in some focus groups as necessary to ensure private sector reporting.

### Conceptual model: opportunities and threats to sustainability

**Figure 2:**
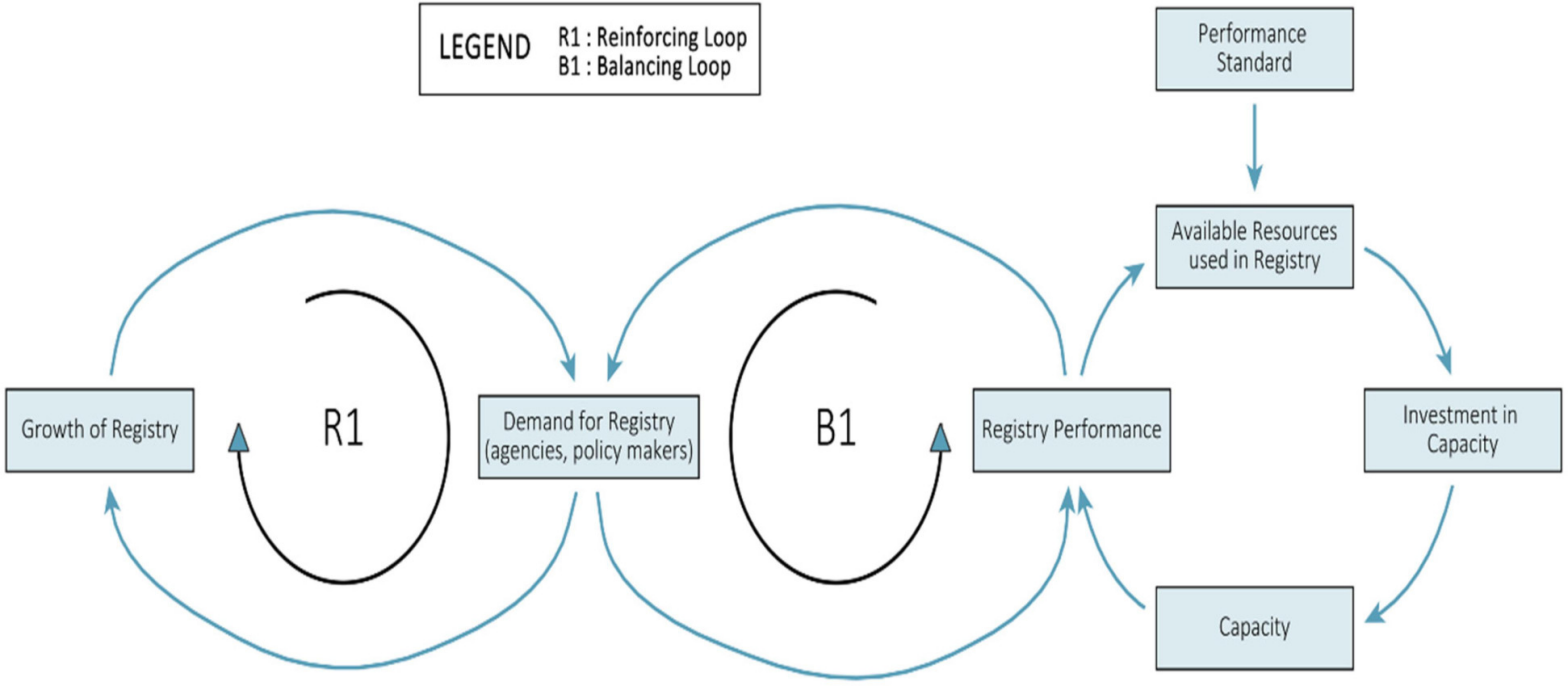
Causal loop diagram highlighting avenues for ensuring sustainable registries.

Despite the regional policies and reported leadership enthusiasm for the development of registries, themes from focus groups indicated the lack of human resource capacity, information technology (IT) infrastructure and funding to support the strengthening of NCD registries. The formal and informal relations within the inner setting (health ministries and care facilities) were important drivers of perceived registry readiness for implementation. The emerging systems archetype (Figure 2) indicated that while demand for registry will lead to its growth, attempts to operationalize a registry without adequate resources may lead to sub-par performance and negatively influence demand for registry data.

Given the challenges identified in achieving operationalisation, we propose a set of minimum inputs and activities required to improve feasibility and readiness of a chronic disease registry in small island developing states (Table 2). These actions were discussed with and received input from the countries involved in the focus groups. Countries that revealed greater political support and had identified a focal point in the focus groups were more likely to report confidence in successful registry development. Focus groups participants identified specific data management training needs reflected in minimum set of activities (Table 2).

**Table 2:** Proposed set of inputs and minimum set of activities based on emerging themes.

Inputs	Minimum set of activities
Individuals • Strong, motivated leadership • Trained registry team	• Key stakeholder engagement • Feasibility and readiness assessment and registry prioritization • Development of implementation plan • Standardized patient and data flow assessment • Capacity building on registry science and operation • Development registry manual • Data management • Data quality training
Inner Setting • Functional Electronic Medical Records • IT Hardware and Software • Sustained Funding	
Outer Setting • Political support • Supportive legal framework • Regional/International technical support (e.g., capacity building, standard operating procedures development	
Inner and outer Setting • Public-private sector cooperation/communication	

## Discussion

Our study highlights the complex interactions between inner and outer settings of NCD registry development and their interplay with individual characteristics in the assessment of readiness and feasibility as examined against elements that have been identified as necessary for successful registry development. Despite calls to strengthen data systems and studies demonstrating the use of data derived from registries and in some cases the benefits of registries, the literature is sparse on describing feasibility and readiness of low resourced settings for registry development.

We combined perspectives from implementation science with a systems thinking approach to better understand the factors affecting the adoption of a complex intervention and to ultimately identify leverage points for action. We used implementation science to evaluate factors influencing registry development and created a causal loop diagram based on the “shifting the burden” archetype to highlight resources needed to strengthen capacity for registry development and maintenance, particularly in low resources settings. By using the “Shifting the Burden” archetype in the development of chronic disease registries, healthcare systems can better rationalise the need to allocate resources and focus on sustainable, root-cause-orientated, evidence-based approaches to chronic disease management.

There is a need for the formation of registry networks to combat challenges related to limited resources (human and financial) in small island developing states. The Caribbean Public Health Agency led the formation of a cancer registry network prior to the COVID-19 pandemic (23). SIDs would benefit from the further development of similar networks for other Non-Communicable diseases. The lack of staff capacity and funding in this study as barriers to the implementation of registries were also identified by findings from Lazem et al. (20). Within resource limited settings this is even more critical to successful implementation. Capacity building provided through registry networks in the form of training workshops and process development have been shown to contribute to successful establishment of registries (24). CARPHA's Cancer Registry Network created a data manual and standard operating procedures that countries have adopted and adapted (25).

### Strengths and limitations

This work provides an in-depth view into health information system dynamics in small island developing states showcasing barriers and facilitators of implementing a data-focused intervention (e.g., registry) that can use real-world data generated from within the health system to inform policy and practice. The methods used could be strengthened by the addition of another data collection method like an audit or documentary analysis that would give further data for triangulation. We used the CFIR framework to inform questionnaire development and data analysis. During our study, a new CFIR framework was released into the peer-reviewed literature and even though we attempted to revise the analysis based on this, the questionnaire had already been developed. This had implications for the way we eventually analysed the readiness construct which was not present in CFIR 2.0 (26).

There was high interest at central government levels in NCD registry development, but infrastructural and human resource capacity barriers likely contribute to a sub-optimal implementation in all territories. In SIDS, we recommend greater collaboration between countries and enhanced regional support to overcome some of the human resource capacity challenges that may impede implementation. Registries are complex interventions into a complex system and more work is needed to understand factors affecting feasibility, readiness and successful implementation in countries that require these interventions to improve NCD management, monitoring and evaluation.

### Reflexivity

As researchers, we met weekly and discussed the feasibility of developing a chronic disease registry in the territories that participated. The primary concern was the success of the project within the time frame outlined by the funders. In addition to using the frameworks identified, our decisions were shaped by our experience in building and maintaining other registries like the Barbados National Registry for Non-Communicable Diseases.

## Conclusion

Small island developing states face unique challenges in preventing and managing Non-Communicable diseases and mental health conditions. The small population sizes create challenges in obtaining sufficient quality staff to fulfil the tasks potentially required for multiple disease registries. The multiple roles of individuals and reliance on single individuals significantly impacts readiness, feasibility and the ability to adopt and sustain registries. While the human resource challenges in this setting are well documented, the extent to which respondents regarded the need for external organizations was instructive. We developed a feasibility assessment tool and proposed a critical set of inputs and activities needed for individuals, inner and outer setting to successfully implement a disease registry in low-resourced settings. Further research is needed to determine the usefulness of this tool in other SIDs.

## Data Availability

The raw data supporting the conclusions of this article will be made available by the authors, without undue reservation.

## References

[1] World Health Organization. Non-communicable Disease and Mental Health in Small Island Developing States. Geneva: World Health Organization (2023).

[2] Etienne CF. Ten years of the port of Spain declaration: lessons learned from tackling noncommunicable diseases in the Caribbean. Rev Panam Salud Publica. (2018) 42:e107. 10.26633/RPSP.2018.107 31093135 PMC6386143

[3] Loft P Parkin EAG. Healthcare in the overseas territories and access to UK care (house of commons research briefing 9802). (2023).

[4] World Health Organization. POLICY BRIEF: Noncommunicable Diseases and Mental Health Conditions in SIDS. Geneva: World Health Organization (2021).

[5] AHRQ Methods for Effective Health Care. In: Gliklich RE Dreyer NA Leavy MB, editors. Registries for Evaluating Patient Outcomes: A User’s Guide. Rockville, MD, United States: Agency for Healthcare Research and Quality (US) (2014). p. 13–9. 24945055

[6] Fonarow GC Zhao X Smith EE Saver JL Reeves MJ Bhatt DL Door-to-needle times for tissue plasminogen activator administration and clinical outcomes in acute ischemic stroke before and after a quality improvement initiative. JAMA. (2014) 311(16):1632–40. 10.1001/jama.2014.3203 24756513

[7] Ormseth CH Sheth KN Saver JL Fonarow GC Schwamm LH. The American Heart Association’s get with the guidelines (GWTG)-stroke development and impact on stroke care. Stroke Vasc Neurol. (2017) 2(2):94–105. 10.1136/svn-2017-000092 28959497 PMC5600018

[8] Giampaoli S Hammar N Adany R De Peretti C, Group ER. Population-based register of stroke: manual of operations. Eur J Cardiovasc Prev Rehabil. (2007) 14(3):S23–41. 10.1097/01.hjr.0000277987.10473.6f 18091133

[9] Madsen M Gudnason V Pajak A Palmieri L Rocha EC Salomaa V Population-based register of acute myocardial infarction: manual of operations. Eur J Cardiovasc Prev Rehabil. (2007) 14(3):S3–22. 10.1097/01.hjr.0000277986.33343.94 18091134

[10] Zaletel M Kralj M. METHODOLOGICAL guidelines and Recommendations for Efficient and Rational Governance of Patient Registries [Electronic Source]. Ljubljana: Slovenia National Institute of Public Health (2015). Available online at: https://health.ec.europa.eu/document/download/bf9d3595-e39a-4c6d-abb3-489990b08365_en

[11] Kroll M Phalkey RK Kraas F. Challenges to the surveillance of non-communicable diseases–a review of selected approaches. BMC Public Health. (2015) 15:1243. 10.1186/s12889-015-2570-z 26672992 PMC4682212

[12] Mandavia R Knight A Carter AW Toal C Mossialos E Littlejohns P What are the requirements for developing a successful national registry of auditory implants? A qualitative study. BMJ Open. (2018) 8(9):e021720. 10.1136/bmjopen-2018-021720 30209155 PMC6144326

[13] Rose AM Hambleton IR Jeyaseelan SM Howitt C Harewood R Campbell J Establishing national noncommunicable disease surveillance in a developing country: a model for small island nations. Rev Panam Salud Publica. (2016) 39(2):76–85. PMID: . 27754515

[14] Whelan J Fraser P Bolton KA Love P Strugnell C Boelsen-Robinson T Combining systems thinking approaches and implementation science constructs within community-based prevention: a systematic review. Health Res Policy Syst. (2023) 21(1):85. 10.1186/s12961-023-01023-4 37641151 PMC10463953

[15] Chamberlain P Brown CH Saldana L. Observational measure of implementation progress in community based settings: the stages of implementation completion (SIC). Implement Sci. (2011) 6:116. 10.1186/1748-5908-6-116 21974914 PMC3197550

[16] Harris PA Taylor R Minor BL Elliott V Fernandez M O'Neal L The REDCap consortium: building an international community of software platform partners. J Biomed Inform. (2019) 95:103208. 10.1016/j.jbi.2019.103208 31078660 PMC7254481

[17] Harris PA Taylor R Thielke R Payne J Gonzalez N Conde JG. Research electronic data capture (REDCap)–a metadata-driven methodology and workflow process for providing translational research informatics support. J Biomed Inform. (2009) 42(2):377–81. 10.1016/j.jbi.2008.08.010 18929686 PMC2700030

[18] Damschroder LJ Aron DC Keith RE Kirsh SR Alexander JA Lowery JC. Fostering implementation of health services research findings into practice: a consolidated framework for advancing implementation science. Implement Sci. (2009) 4:50. 10.1186/1748-5908-4-50 19664226 PMC2736161

[19] California HealthCare Foundation. California physician orders for life-sustaining treatment (POLST) ERegistry readiness assessment tool. (2018). Available online at: https://capolst.org/wp-content/uploads/2019/08/RegistryReadinessAssessment.pdf (Accessed June 12, 2023).

[20] Lazem M Sheikhtaheri A. Barriers and facilitators for the implementation of health condition and outcome registry systems: a systematic literature review. J Am Med Inform Assoc. (2022) 29(4):723–34. 10.1093/jamia/ocab293 35022765 PMC8922163

[21] Hennink M Kaiser BN. Sample sizes for saturation in qualitative research: a systematic review of empirical tests. Soc Sci Med. (2022) 292:114523. 10.1016/j.socscimed.2021.114523 34785096

[22] Kim D. Systems archetypes I: diagnosing systemic issues and designing high-leverage interventions. (2000). Available online at: https://thesystemsthinker.com/systems-archetypes-i-diagnosing-systemic (Accessed January 11, 2025).

[23] Caribbean Public Health Agency. IARC caribbean cancer registry hub trinidad and tobago. (2021). Available online at: https://caribbeancrh.carpha.org/ (Accessed August 10, 2025).

[24] Yarney J Ohene Oti NO Calys-Tagoe BN Gyasi RK Agyeman Duah I Akoto-Aidoo C Establishing a cancer registry in a resource-constrained region: process experience from Ghana. JCO Glob Oncol. (2020) 6:610–6. 10.1200/JGO.19.00387 32302237 PMC7193799

[25] Caribbean Public Health Agency. Caribbean Registry Manual: Data Collection and Operating Procedures Module. Trinidad and Tobago: CARPHA (2018).

[26] Damschroder LJ Reardon CM Widerquist MAO Lowery J. The updated consolidated framework for implementation research based on user feedback. Implement Sci. (2022) 17(1):75. 10.1186/s13012-022-01245-0 36309746 PMC9617234

