# Supplementary material for: Complex inner and outer setting interactions determine feasibility and readiness of developing primary care registries in small island developing states: sequential mixed methods study
Source: Front Health Serv. 2025 Sep 30;5:1593902. doi: 10.3389/frhs.2025.1593902 (PMC12518279; doi:10.3389/frhs.2025.1593902)
Supplement: Supplementary file 1 [file Table1.docx]

Supplemental material

Supplemental Table 1- CFIR domain structure and associated constructs for Feasibility and Readiness Assessments.

| **Feasibility assessment constructs** | | | **Readiness assessment constructs** | |
| --- | --- | --- | --- | --- |
| **CFIR Domain: Intervention Characteristics** | | | | |
| - Disease of interest - Registry’s purpose | | - Target population - Resources | - Available Resources | |
| **CFIR Domain: Outer setting** | | |  | |
| - External policies | - Connectivity - Data legislation | | - Vital statistics department | - Scope |
| - Scope (e.g. private sector, laboratories) - IT infrastructure management | | | - Data legislation |  |
| **CFIR Domain: Inner Setting** | | |  | |
| - EHR functionality - Data availability | | - Build registry team - Staff capacity | - Build registry team - Staff capacity | |
| - Networks & communications - HIT & data exchange capacity | | | - Networks & communications - Readiness for implementation | |
| - Readiness for implementation | | |  | |
| **CFIR Domain: Characteristics of indviduals** | | | | |
| - Knowledge & beliefs about intervention - Self-efficacy | | | - Knowledge & beliefs about the Intervention - Self-efficacy - Leadership engagement | |

Supplemental Table 2: Constructs identified from CFIR*, AHRQ** user’s guide for registries for evaluating patient outcomes (AHRQ) and the Physician Orders for Life-Sustaining Treatment (POLST) registry readiness assessment

| **Constructs** | **Description** | | **Construct origin** | | | | | |
| --- | --- | --- | --- | --- | --- | --- | --- | --- |
| Intervention Characteristics |  | | | | |  | | |
| Intervention source | Who developed the intervention and why is it being implemented | | | | | CFIR | | |
| Adaptability | Potential alterations to the intervention for successful implementation | | | | | CFIR | | |
| Trialability | Can/will the intervention be trialled | | | | | CFIR | | |
| Complexity | Addresses the complexity of intervention not the implementation | | | | | CFIR | | |
| Design quality & packaging | Perceptions on quality of supporting materials | | | | | CFIR | | |
| Cost | Costs incurred in establishing registry | | | | | CFIR | | |
| Disease(s) of interest | A disease focus is needed for the registry before implementation can start | | | | | AHRQ | | |
| Registry’s purpose | The purpose of the registry guides the choice of core data | | | | | AHRQ | | |
| Core data | Each data element should relate to the purpose of the registry | | | | | AHRQ | | |
| Patient outcomes | Outcomes of greatest importance should be identified early in the concept phase of the registry | | | | | AHRQ | | |
| Target population | The population to which the findings of the registry are meant to apply | | | | | AHRQ | | |
| Protocol development | A protocol will document the objectives, design, participant inclusion/exclusion criteria, outcomes of interest, data to be collected, data collection procedures, governance procedures, and plans for complying with ethical obligations and protecting patient privacy | | | | | AHRQ | | |
| Statistical analysis plan | Allows data elements to be defined, keeps focus on the registry purpose and limits the number of extraneous (“nice to know”) data elements | | | | | AHRQ | | |
| Outer setting |  | | | | |  | | |
| Cosmopolitanism | Networking & information sharing | | | | | CFIR | | |
| Laws & external polices | Legislation, professional group guidelines that support implementation of the registry. Particularly with respect to data handling | | | | | CFIR  POLST | | |
| Governance/oversight plan | Considerations such as data protection, security and sharing | | | | | AHRQ | | |
| Internet speed & reliability | To enable efficient data sharing/collection reliable internet is key | | | | | POLST | | |
| Inner Setting | |  | | | | |  | |
| Structural characteristics | | Potential changes needed to accommodate the intervention. | | | | | CFIR | |
| Networks & communication | | Communications within team and with stakeholders | | | | | CFIR | |
| Culture | | Potential impact of organizational culture on implementation | | | | | CFIR | |
| Implementation climate | | Reception of implementation | | | | | CFIR | |
| Readiness for implementation | | Tangible indicators of organizational commitment to its decision to implement an intervention. | | | | | CFIR | |
| Financing | | Key to sustainability is funding. This can also come from external sources (outer setting) | | | | | CFIR AHRQ | |
| Identify key stakeholders | | Establishment of network of data sources and those involved with registry | | | | | AHRQ | |
| Data availability | | Successful registries are data dependent, ideally electronically | | | | | AHRQ | |
| Build registry team | | Includes core team that manage the registry as well as those involved in the data collection process | | | | | AHRQ | |
| Health Information Technology | | An EHR offers a norm for electronic systems usage within the organization and confirms that electronic data storage and use is standard for staff. | | | | | POLST | |
| Data exchange capacity | | Participation in health information exchange may help to address some of the external data transmission issues | | | | | POLST | |
| EHR functionality | | EHR alone does not guarantee efficient data collection, functionality such as “searchability” and single sign-on are key for efficient use of EHR | | | | | POLST | |
| Staff capacity | | Registry success is dependent on the staff having the capacity to perform registry related tasks | | | | | POLST | |
| Current use of data | | Current use of data in reports highlight the ability for data extraction being used to create reports | | | | | POLST | |
| Characteristics of individuals | |  | | | | |  | |
| Knowledge & beliefs about the intervention | | Individuals’ attitudes toward and value placed on the intervention | | | | | CFIR | |
| Self-efficacy | | Individual belief in their own capabilities to execute courses of action to achieve implementation goals | | | | | CFIR | |
| Individual stage of change | | Characterization of the phase of change an individual is in | | | | | CFIR | |
| Individual identification with organization | | Individuals’ perceptions of the organization, their relationship and degree of commitment with that organization | | | | | CFIR | |
| Other personal attributes | | Other personal traits such as tolerance of ambiguity | | | | | CFIR | |
| Team engagement | | Speed of response, frequency of communication and level of engagement in meetings | | | | | CFIR | |
| Implementation Process | |  | | | | |  |  |
| Planning | | Development of tasks to enable implementation of the intervention | | | | | CFIR |  |
| Engaging stakeholders, clinical & administrative champions | | Involvement of appropriate individuals in implementation & use of intervention | | | | | CFIR POLST  AHRQ |  |
| Executing | | Completing of implementation in accordance with the plan | | | | | CFIR |  |
| Reflecting & evaluating | | Quantitative and qualitative feedback about the progress and quality of implementation | | | | | CFIR |  |
| Assess feasibility | | This includes identifying funding, data collection processes and staff capacity | | | | | AHRQ |  |
| Develop project plan | | Needed to ensure timely implementation of the registry | | | | | AHRQ |  |
|  | |  | | | | |  |  |

Supplemental Table 3: Scoring system for feasibility assessment

| **Constructs** | **Question topics** | | **Scoring**  Shaded boxes indicate core selection criteria | | | | | |
| --- | --- | --- | --- | --- | --- | --- | --- | --- |
| Intervention Characteristics | | |  | | | | | |
| Disease(s) of interest | Top 3 diseases | | No score – but provides information on health priorities | | | | | |
|  | Primary disease of interest | | This response required selection. Each response was considered a “vote” for a particular choice | | | Unanimous choice =2  Majority =1 | | |
| Registry’s purpose | Primary aim of registry | | This response required selection. Each response was considered a “vote” for a particular choice | | | Unanimous choice =2  Majority =1 | | |
| Outer setting |  | |  | | |  | | |
| Target population | Proportion of private primary care | | This response was on a sliding scale. The median of the group response was used to allocate a score | | | Median score less than 10%=2  Median score less than 50%=1  Median score greater than 50%=0 | | |
|  | No. of private hospitals | | Provides estimate of the volume of potentially hard to obtain data | | | | | |
|  | Ease of obtaining data | | This response was on a sliding scale. The median of the group response was used to allocate a score | | Very straightforward to obtain data (median 1 on sliding scale) = 2  Possible to obtain data (median 2/3 on sliding scale) = 1  Data unobtainable (median score from sliding scale 4/5) = 0 | | | |
|  | No. of private pharmacies | | Provides estimate of the volume of potentially hard to obtain data | | | | | |
|  | Ease of obtaining data from private pharmacies | | This response was on a sliding scale. The median of the group response was used to allocate a score | | Very straightforward to obtain data (median 1 on sliding scale) = 2  Possible to obtain data (median 2/3 on sliding scale) = 1  Data unobtainable (median score from sliding scale 4/5) = 0 | | | |
|  | No. of private labs | | Provides estimate of the volume of potentially hard to obtain data | | | | | |
|  | Ease of obtaining data from private laboratories | | This response was on a sliding scale. The median of the group response was used to allocate a score | | Very straightforward to obtain data (median 1 on sliding scale) = 2  Possible to obtain data (median 2/3 on sliding scale) = 1  Data unobtainable (median score from sliding scale 4/5) = 0 | | | |
|  | Identification of hard to access populations | | This response was on a sliding scale. The median of the group response was used to allocate a score | | Proportion of hard to reach populations <10% = 2  <50% = 1  >51% = 0 | | | |
| Laws & external polices | Legislation limiting data sharing | | This response required a yes or no. Each response was considered a “vote” to indicate whether or not there was a policy preventing data sharing. | | | | | Clear indication of policy status = 1  Unclear on policy status=0 |
| Internet speed & reliability | Internet speed | | Provides insight into IT infrastructure and territory wide variation | | | | | |
|  | Internet reliability | | This response required a yes or no. Each response was considered a “vote” to indicate whether or not there was a reliable internet | | | | | Consensus on good reliability =2  Possible good reliability=1  Unreliable or no consensus=0 |
| **Constructs** | | **Question topics** | | **Scoring**  Shaded boxes indicate core selection criteria | | | | |
| Inner Setting | |  | |  | | | | |
| Networks & communication | | Usual communication strategy for new initiatives | | Provides insight into avenues for disseminating information regarding the registry | | | | |
| Readiness for implementation | | Any current or previous registry processes | | Provides insight into avenues for potential data collection | | | | |
| Financing | | Availability of funding | | Although not vital for implementation, funding is key to the sustainability of the registry | | | | |
| Identify key stakeholders | | IT infrastructure management  Access to epidemiologist | | If present these are key stakeholders the registry team need to be working with | | | | |
| Build registry team | | Project lead  Access to epidemiologist | | A project lead is needed to guide the implementation of the registry, while access to an epidemiologist can help with understanding of data collection and analysis | | | | |
| EMR functionality | | Existence of EMR | | EMR can facilitate efficient data collection processes | | | | |
|  |  | Searchable by diagnosis | | This response required a yes or no. Each response was considered a “vote” to indicate whether or not the EMR was searchable by diagnosis | | | | Consensus of “yes” = 1 |
|  |  | Allows data extraction  Possibility of incorporating a registry page into EMR  Links to prescription data  Links to laboratory data | | This EMR functionality is needed for EMR to aid data collection | | | | |
| Staff capacity | | Sufficient time | | This response required a yes or no. Each response was considered a “vote” to indicate whether or not staff would have the time to manage a registry | | | | Majority stated “yes” = 1 |
| Characteristics of individuals | | | |  | | | | |
| Knowledge & beliefs about the intervention | | Previous access to information on registries | | Provides insight into the registry team’s potential knowledge base | | | | |
|  |  | Importance of external technical support | | This response was on a sliding scale. The median of the group response was used to allocate a score | | | Technical support critical for success(score 9/10) = 2  Technical support helpful to success (score 7/8) = 1  Technical support not important (<6) = 0 | |
|  |  | Utility of a registry to improve health care | | This response was on a sliding scale. The median of the group response was used to allocate a score | | | Median >90% = 2  Median 70-80% = 1  Median < 70% =0 | |

| **Constructs** | **Question topics** | **Scoring**  Shaded boxes indicate core selection criteria | |
| --- | --- | --- | --- |
| Characteristics of individuals continued | | | |
| Self-efficacy | Confidence in successful registry implementation | This response was on a sliding scale. The median of the group response was used to allocate a score | Median >90% = 2  Median 70-80% = 1  Median < 70% =0 |
| Team engagement | Timely response to survey completion, information requests & engagement^[[1]](#endnote-1)^ | This score was based on time to respond to information & survey requests as well as qualitative assessments of engagement | Full engagement=3  Partial engagement=2  Limited engagement =1 |

Supplemental Table 4: Theory based coding framework: An exploration of inner setting – facilitators and barriers of registry implementation

| **Coding name** | **Abbreviation** | **Definition- refers to all references in documentary sources relating to** |
| --- | --- | --- |
| Structural Characteristics  1. Physical Infrastructure  2. Information Technology Infrastructure  3. Work Infrastructure | SC  SC_PI  SC_ITI  SC_WI | Infrastructure components support functional performance of the Inner Setting  Layout and configuration of space and other tangible material features support functional performance of the Inner Setting  Technological systems for tele-communication, electronic documentation, and data storage, management, reporting, and analysis support functional performance of the Inner Setting  Organization of tasks and responsibilities within and between individuals and teams, and general staffing levels, support functional performance of the Inner Setting. |
| Communications | Comm | There are high quality formal and informal information sharing practices within and across Inner Setting boundaries (e.g., structural, professional) |
| Relational Connections | Rel_Con | There are high quality formal and informal relationships, networks, and teams within and across Inner Setting boundaries (e.g., structural, professional) |
| Culture | Cul | There are shared values, beliefs, and norms across the Inner Setting  *Use this construct to capture themes related to Culture that are not included in the subconstructs below* |
| Culture (extended/explained)  1.Human Equality-Centeredness  2. Recipient-Centeredness  3. Deliverer-Centeredness  4. Learning-Centeredness | Hum_eq  Recip_c  Deliv_c  Learn_c | There are shared values, beliefs, and norms about the inherent equal worth and value of all human beings  There are shared values, beliefs, and norms around caring, supporting, and addressing the needs and welfare of recipients  There are shared values, beliefs, and norms around caring, supporting, and addressing the needs and welfare of deliverers  There are shared values, beliefs, and norms around psychological safety, continual improvement, and using data to inform practice |
| E. Tension for Change | Ten_chan | The current situation is intolerable and needs to change |
| F. Compatibility | Compat | The innovation fits with workflows, systems, and processes |
| G. Relative Priority | Rel_pri | Implementing and delivering the innovation is important compared to other initiatives |
| H. Incentive Systems | Incen | Tangible and/or intangible incentives and rewards and/or disincentives and  punishments support implementation and delivery of the innovation |
| I. Mission Alignment | Mis_ali | Implementing and delivering the innovation is in line with the overarching commitment, purpose, or goals in the Inner Setting |
| J. Available Resources  1. Funding  2. Space  3. Materials & Equipment | Av_res | Resources are available to implement and deliver the innovation  Funding is available to implement and deliver the innovation  Physical space is available to implement and deliver the innovation  Supplies are available to implement and deliver the innovation |
| K. Access to Knowledge & Information | Know_inf | Guidance and/or training is accessible to implement and deliver the innovation. |

1. This construct also has a qualitative component based on interactions with the team both verbally and in writing. [↑](#endnote-ref-1)
