# Supplementary material for: Complex inner and outer setting interactions determine feasibility and readiness of developing primary care registries in small island developing states: sequential mixed methods study
Source: Front Health Serv. 2025 Sep 30;5:1593902. doi: 10.3389/frhs.2025.1593902 (PMC12518279; doi:10.3389/frhs.2025.1593902)
Supplement: Supplementary file 2 [file Table2.docx]

Table 1: Survey responses by territory

| **Questions within CFIR Domains** | **C1 (n=8)** | | | **C2 (n=8)** | | | **C3 (n=10)** | | | **C4 (n=10)** | | |
| --- | --- | --- | --- | --- | --- | --- | --- | --- | --- | --- | --- | --- |
| **Domain: Intervention characteristics** |  |  |  |  |  |  |  |  |  |  |  |  |
| Primary Disease (Top 3) | Hypertension (4) | Diabetes (2) | CVD (2) | Cancer (4) | Diabetes (3) | Hypertension (1) | Diabetes (3) | Cancer (3) | Hypertension (1) | Diabetes (7) | Obesity (2) | Chronic Kidney Disease (1) |
| Purpose | Monitoring and Improving Care (8) |  |  | Monitoring & improving care (4) | Disease burden monitoring (2) | Outcomes monitoring (2) | Monitoring & improving care (4) | Disease burden monitoring (3) | Indicator tracking (2) | Monitoring & improving care (8) | Disease burden monitoring (1) | Outcomes monitoring (1) |
| Utility of a registry for improving healthcare | Range 85-100 | Median 93.5 |  | Range 70-100 | Median 93 |  | Range 75-100 | Median 94 |  | Range 79-100 | Median 91 |  |
| Confidence in successful registry implementation | Range 50-100 | Median 89 |  | Range 34-97 | Median 80.5 |  | Range 50-90 | Median 75 |  | Range 10-100 | Median  60 |  |
| **Domain: Individual characteristics** |  |  |  |  |  |  |  |  |  |  |  |  |
| Access to epidemiologist | Yes=6 | Unsure=2 |  | Yes=7 | No =1 |  | Yes=10 |  |  | Yes= 2 | No = 5 | Unsure= 3 |
| Location of epidemiologist | Internal=6 |  |  | Internal = 7 |  |  | Internal=7 | External=3 |  | External = 2 |  |  |
| Has a lead been identified | Yes=3 | No=2 | Unsure=3 | Yes=3 | No=3 | Unsure=2 | No=1 | Unsure=9 |  | Yes =2 | No =2 | Unsure= 6 |
| **Domain: Inner Setting** |  |  |  |  |  |  |  |  |  |  |  |  |
| IT management | In-house=3 | Externally= 2 | Unsure=3 | In-house =6 | External =1 | Unsure=1 | In-house =2 | Externally=3 | Unsure=5 | In-house = 6 | Externally =3 | Unsure =1 |
| Internet reliability | Very reliable= 3 | Reliable = 3 | Not reliable = 2 | Very reliable =3 | Reliable =3 | Not reliable=2 | Very reliable=2 | Reliable=4 | Not reliable=3 | Reliable=6 | Not reliable=4 |  |
| Internet connection speed | Very fast = 4 | Fast = 2 | Average = 2 | Very fast =3 | Fast  = 1 | Average =4 | Very fast=1 | Fast=4 | Average=4 | Average =7 | Poor= 3 |  |
| EHR/EMR system | Yes= 8 |  |  | Yes= 8 |  |  | Yes=6 | No=3 |  | Yes= 9 |  |  |
| Which levels of health care have EHR/EMR? | Primary= 7 | Secondary =7 | Tertiary= 2 | Primary =7 | Secondary =7 | Tertiary= 3 | Primary=3 | Secondary=4 | Unsure =2 | Primary= 8 | Secondary=6 | Tertiary=2 |
| Do they all use the same system? | Yes=7 |  |  | Yes= 2 | No= 4 |  | Yes=3 |  |  | Yes=2 | No=4 |  |
| Data extraction from EHR/EMR? | Yes= 6 | Unsure= 2 |  | Yes=7 | No=1 |  | Yes=4 | Unsure=2 |  | Yes=5 | No=2 | Unsure=2 |
| EHR searchable by diagnosis | Yes= 3 | Unsure = 4 |  | Yes=6 | Unsure =2 |  | Yes=1 | Unsure=5 |  | Yes=1 | No=5 | Unsure= 3 |
| Registry form incorporated into EHR | Yes= 4 | Unsure =4 |  | Yes=4 | Unsure= 3 |  | Yes=1 | Unsure =5 |  | Yes= 2 | Unsure = 7 |  |
| EHR linked to prescription data | Yes= 5 | Unsure = 1 |  | Yes =8 |  |  | Yes= 2 | Unsure=4 |  | Yes=9 |  |  |
| Physical space | Yes= 6 | No =1 | Unsure=1 | Yes = 6 | Unsure =2 |  | Yes=5 | Unsure= 5 |  | Yes= 4 | No= 3 | Unsure=3 |
| Time | Yes=4 | Unsure=4 |  | Yes = 7 | Unsure = 1 |  | Yes=6 | Unsure=4 |  | Yes= 2 | No= 2 | Unsure=6 |
| Financial resources | Yes=1 | No=2 | Unsure=5 | Yes =4 | No = 2 | Unsure=2 | Yes= 4 | Unsure=6 |  | Yes=2 | No=2 | Unsure=6 |
| **Domain: Outer Setting** |  |  |  |  |  |  |  |  |  |  |  |  |
| Any hard-to-reach populations | Yes=3 | No=3 | Unsure=2 | Yes=6 | No=1 | Unsure=1 | Yes=3 | No=1 | Unsure=6 | No=7 | Unsure= 2 |  |
| Proportion of hard to reach * | Range NA | Median 32 |  | Range 12-83 | Median 30 |  | Range 11-26 | Median 25 |  | No response |  |  |
| Who are hard to reach | Those seeking care overseas |  |  | Latinx | From private clinics or seeking care overseas | Undocumented patients | Those who do not access public health care services | Persons who attend private doctor or seek medical assistance overseas |  | No response |  |  |
| Policies preventing EHR data sharing | Yes=2 |  |  | Yes= 2 | No =6 |  | Yes= 1 | Unsure=6 |  | Yes= 5 | No= 3 | Unsure= 2 |
| Proportion of PPHC | Range 15-50 | Median 27.5 |  | Range 50- 80 | Median 63 |  | Range 0 - 50 | Median 20 |  | None |  |  |
| Private hospitals (PH) | Yes=4 | No=4 |  | Yes= 7 | No = 1 |  | No=10 |  |  | No=10 |  |  |
| Ease of data collection from PH | Range 2-4 | Median 4 |  | Range 1-5 | Median 3 |  | Not applicable |  |  | Not applicable |  |  |
| Private pharmacies (PP) | Yes = 8 |  |  | Yes= 8 |  |  | Yes = 10 |  |  | No=10 |  |  |
| Ease of data collection from PP | Range 3-4 | Median 3 |  | Range 2-5 | Median 3 |  | Range 1-5 | Median 3 |  | Not applicable |  |  |
| Private laboratories (PL) | Yes= 8 |  |  | Yes=8 |  |  | Yes=10 |  |  | No= 10 |  |  |
| Ease of data collection from PL | Range 2-4 | Median 3.5 |  | Range 1-5 | Median 3 |  | Range 1-4 | Median 3 |  | Not applicable |  |  |
| Need for external technical support | Yes= 8 |  |  | Yes= 6 | No=1 | Unsure=1 | Yes=10 |  |  | Yes= 9 | Unsure = 1 |  |
| Importance of technical support | Range 5-10 | Median 9 |  | Range 1-9 | Median 7 |  | Range 5-10 | Median 8 |  | Range 6-10 | Median 8 |  |
